# The prognostic role of IDH mutations in homogeneously treated patients with anaplastic astrocytomas and glioblastomas

**DOI:** 10.1186/s40478-019-0817-0

**Published:** 2019-10-17

**Authors:** Arne Christians, Antonia Adel-Horowski, Rouzbeh Banan, Ulrich Lehmann, Stephan Bartels, Felix Behling, Alonso Barrantes-Freer, Christine Stadelmann, Veit Rohde, Florian Stockhammer, Christian Hartmann

**Affiliations:** 1 Department of Neuropathology, Institute of Pathology, Hannover Medical School (MHH), Carl-Neuberg-Str. 1, D-30625 Hannover, Germany; 2 Department of Neurosurgery, University Medical Center Göttingen, Göttingen, Germany; 3 Department of Child and Adolescent Psychiatry, Charité Universitätsmedizin, Berlin, Germany; 4 Institute of Pathology, Hannover Medical School, Hannover, Germany; 5 Department of Neurosurgery, University Hospital Tübingen, Tübingen, Germany; 6 Institute of Neuropathology, University Medical Center Göttingen, Göttingen, Germany; 7 Department of Neuropathology, Institute of Pathology, University Medical Center Leipzig, Leipzig, Germany; 8 Department of Neurosurgery, Städtisches Klinikum Dresden, Dresden, Germany

**Keywords:** IDH1, IDH2, MGMT, EGFR, Prognosis, Grading, Anaplastic astrocytomas, Glioblastomas

## Abstract

The detection of IDH mutations in patients with diffusely infiltrating malignant astrocytomas resulted in substantial modifications in the concept of WHO classification of these tumors. An important underlying observation was that patients with anaplastic astrocytomas (AA) without IDH mutation had a clinical course similar to that of patients with glioblastomas (GBM). The underlying observations of the German Glioma Network and NOA-04, however, were based on mixed patient cohorts. While most GBM patients received combined radiochemotherapy, patients with AA usually had radiotherapy or chemotherapy only. This intrinsic shortcoming of the study raised the question of whether patients with AA, IDH wildtype, WHO grade III, might have better prognosis if treated with combined radiochemotherapy than patients with GBM receiving the same combination therapy. Thus, the question remains whether the established histopathological grading criteria for malignant astrocytomas in the absence of an IDH mutation are still important if neither vascular proliferation nor necrosis are detectable. All patients in the cohort investigated here with the diagnosis of AA or GBM were subjected to a combined radiochemotherapy according to the Stupp protocol independently of the histopathological diagnosis. Thus, the analysis of these patients allows to clarify whether patients with AA, IDH wildtype, WHO grade III have a prognosis similar to that of GBM, IDH wildtype, WHO grade IV, even under equivalent therapeutic conditions. We determined the *IDH1* and *IDH2* status by sequencing, the *MGMT* status by pyrosequencing after bisulfite treatment and the *EGFR* status of the patients by FISH. In fact, the patients with the histopathological diagnosis of an AA IDH wild-type under similar aggressive therapy showed a comparable and therefore no better prognosis (median overall survival (mOS) 16 months) than patients with a GBM (mOS 13 months). Instead, patients with an AA and an IDH mutation receiving the same therapy had a mOS of 54 months. Thus, it can be concluded that in the absence of an IDH mutation, the established histopathological grading criteria ‘necrosis’ and ‘vascular proliferation’ actually lose their prognostic significance. If, on the other hand, patients with malignant astrocytomas and an IDH mutation are examined, there is still a difference between patients with necrosis and/or vascular proliferation and those whose tumors do not show such characteristics. Accordingly, in patients with malignant astrocytomas with IDH mutation it can be concluded that a histological differentiation between AA IDH mutated and GBM IDH mutated remains beneficial from a prognostic perspective.

## Introduction

Diffusely infiltrating malignant astrocytomas are differentiated into anaplastic astrocytomas WHO grade III (AA) and glioblastomas WHO grade IV (GBM) due to the negative prognostic significance of the histopathological biomarkers ‘necrosis’ [1] and ‘microvascular proliferation’ [2] in all editions of the WHO classification of brain tumors [3–7]. According to the revised fourth edition of the WHO classification of brain tumors from 2016, malignant astrocytomas are furthermore differentiated based on their IDH status into the group of anaplastic astrocytomas WHO grade III with/without IDH mutation (AA IDH-mut/AA IDH-wt) and into the group of glioblastomas WHO grade IV with/without IDH mutation (GBM IDH-mut/GBM IDH-wt) [6]. This differentiation of tumors according to IDH status was based on the observation that a positive IDH status correlated with a better prognosis for the patients both within the group of AA [8] and within the group of GBM [9]. Furthermore, it could be demonstrated that patients with AA IDH-wt had a worse prognosis than patients with GBM IDH-mut [10]. Genetically and epigenetically it was also observed that AA and GBM with/without IDH mutation are two different entities. It emerged that most histopathologically defined AA IDH-wt had the epigenetic and genetic signature of GBM IDH-wt. Morphologically diagnosed GBM IDH-mut, on the other hand, were genetically characterized as tumors from the group of IDH mutated diffuse astrocytomas [11–14]. For example, GBM IDH-wt carry *EGFR* amplifications in around 45% [15] and *TERT* mutations in up to 80% [16] while astrocytomas with an IDH mutation exhibit such alterations in a rather low number [12].

Patients with GBM show a significantly better response to chemotherapy with alkylating substances such as temozolomide when the *MGMT* promoter is methylated [17]. The detection of an IDH mutation correlates with *MGMT* methylation: almost all patients with an IDH mutation also show *MGMT* promoter methylation [18]. The link between IDH mutations and *MGMT* promoter methylation is presumably due to the competitive inhibition of the alpha-ketogluterate-dependent dioxygenase TET2 by the oncometabolite-2′-hydroxgluterate. As a result, a global hypermethylation of the genome of tumor cells with an IDH mutation occurs [19] and the gCIMP genotype becomes detectable [20]. In this context, it should be noted that the *MGMT* promoter is also subject to this global hypermethylation [18]. In the absence of an IDH mutation, however, *MGMT* promoter methylation is found in about 40% of all GBM patients. In patients with AA IDH-wt, methylation of the *MGMT* promoter is observed with approximately the same frequency. Accordingly, the *MGMT* status predicts the presumed success of chemotherapy with temozolomide only in those patients with AA IDH-wt [18].

According to the EANO guideline for the treatment of patients with diffuse gliomas [21], combined temozolomide and radiotherapy followed by temozolomide maintenance treatment is recommended upon diagnosis of GBM regardless of IDH status at age under 70 years, the so-called ‘Stupp protocol’ [22]. Patients with a AA IDH-mut are advised to receive primary radiotherapy followed by temozolomide or PCV maintenance treatment. In patients diagnosed with AA IDH-wt, however, radiotherapy alone is recommended for those patients with a negative *MGMT* methylation status and the GBM protocol is recommended for patients with a positive *MGMT* methylation status.

One of the main observations that led to the concept of different therapeutic recommendations is the missing significant prognostic difference between patients with GBM and AA when both entities are defined by the absence of IDH mutation [10]. However, due to the retrospective concept of this trial, GBM patients treated with the more aggressive Stupp regimen were compared with AA patients who received mostly chemotherapy or radiotherapy alone. The question remains whether patients with IDH wild-type malignant astrocytomas without histological evidence of necrosis and/or vascular proliferation, i.e. with AA IDH-wt, do not differ prognostically under the same therapy from patients diagnosed with GBM IDH-wt. This raises the question whether the established histopathological criteria for grading of AA and GBM in the absence of IDH mutations still have a relevant prognostic significance. Furthermore, it could be assumed that patients with AA IDH-wt live longer as a result of a more aggressive treatment according to the Stupp protocol than patients who received predominantly a monotherapy, as the patients of the cohort studied so far [10]. In such a scenario, it would have to be concluded that the histopathological grading criteria for the differentiation of malignant astrocytic tumors are still valid independent of the IDH status.

Patients diagnosed with AA and GBM at the University Medical Center in Göttingen received the same adjuvant treatment recommendations for several years. Thus, this patient cohort is ideal for a multivariate analysis of prognostic factors especially regarding the question if histopathological detection of prominent microvascular proliferation and/or necrosis still have a prognostic significance in times of molecular IDH diagnostics.

## Material and methods

### Patient collective

In this retrospective analysis, all patients with the diagnosis of a AA or GBM were identified who i.) underwent surgery at the Department of Neurosurgery at the University Medical Center Göttingen between 1997 and 2011, ii.) presented with an initial lesion, iii.) received adjuvant radiation therapy and chemotherapy with temozolomide, iv.) had clinical follow-up data and v.) had sufficient formalin-fixed paraffin embedded (FFPE) tumor tissue available for further investigations. Age, preoperative Karnofsky Performance Scale (KPS), extent of resection (EOR), overall survival (OS) were retrieved from the medical records. OS was the primary endpoint of the analysis. FFPE tumor tissue of each tumor was collected from the biobank of the Institute of Neuropathology of the University Medical Center Göttingen. Three experienced neuropathologists (CS, CH, RB) re-evaluated the samples according to the recent definition criteria of the WHO classification [6]. The study was approved by the Ethics Committee of the University Medical Center Göttingen. Cases with incomplete medical datasets or insufficient amounts of viable tumor tissue for further tissue analysis were excluded from the study.

### DNA extraction

FFPE tissue samples were cut into 11 serial sections each. One section per set was subsequently H&E stained. The stained tissue sections were evaluated by an experienced neuropathologist (RB) and areas consisting of at least 80% tumor cells were marked. The corresponding areas on the unstained sections were then scraped into sample tubes, deparaffinized and subjected to DNA extraction with the DNeasy Blood & Tissue Kit (Qiagen, Hilden, Germany). DNA concentration of the resulting solution was quantified using a FLUOstar Omega plate reader photometer (BMG Labtech GmbH, Ortenberg, Germany).

### IDH1 and IDH2 mutation analysis

The *IDH1* and *IDH2* mutation status was determined by Sanger sequencing. PCR amplification of the target region was performed by mixing 50 ng of extracted tumor DNA as template, 10 μl of HotStar Taq 2X Mastermix (Qiagen), 1 μl of the respective forward and reverse primers (10 pmol each) and high purity water to a final volume of 20 μl (PCR primers - *IDH1* forward: CGGTCTTCAGAGAAGCCATT, *IDH1* reverse: GCAAAATCACATTATTGCCAAC, *IDH2* forward: AGCCCATCATCTGCAAAAAC, *IDH2* reverse: CTAGGCGAGGAGCTCCAGT). The PCR cycling protocol consisted of an initial activation step at 95 °C for 5 min, followed by 30 cycles of denaturation at 95 °C for 30 s, annealing at 60 °C for 30 s and elongation at 72 °C for 60 s, concluding with a final elongation step at 72 °C for 10 min. The PCR products were checked by agarose gel electrophoresis. Five microliters of PCR product were mixed with one microliter of Illustra ExoProStar One-Step enzyme mix (GE Healthcare, Buckinghamshire, UK) and incubated at 37 °C for 20 min, followed by inactivation at 80 °C for 15 min. The samples were then mixed with 6 μl of *IDH1*/2 forward primers, respectively, and sent to an external sequencing service provider (GATC, Konstanz, Germany).

### MGMT analysis

The *MGMT* promoter methylation status was determined by pyrosequencing [23]. The tumor DNA was subjected to bisulfite conversion using the EZ DNA Methylation-Gold Kit (Zymo Research, Irvine, CA, USA). The bisulfite converted DNA was then used as PCR template. For PCR amplification, 100 ng of bisulfite converted DNA was added with 0.1 μl Platinium Taq Polymerase (Thermo Fisher Scientific, Waltham, MA, USA), 1 μl of each PCR primer (10 pmol), 1 μl 10 mM dNTP (Thermo Fisher Scientific), 2,5 μl PCR buffer, 2,5 μl MgCl2 (25 mM) and high purity water to a final volume of 25 μl. The PCR primers MGMT_1i5’: GTTTYGGATATGTTGGGATAG, MGMT_3i3’: AACCACTCRAAACTACCACC, MGMT_3i3’bio: Biotin-AACCACTCRAAACTACCACC and MGMT_1i5’bio: Biotin-GTTTYGGATATGTTGGGATAG were used. Because MGMT PCR primers span CpG sites that can be modified through the bisulfite conversion the primer solution consists of an equimolar mix of oligonucleotides with C/T (Y) or G/A (R) nucleotides at the position of the CpG sites. The PCR cycling program was composed of an initial activation step at 95 °C for 5 min, followed by 45 cycles of denaturation at 95 °C for 30 s, annealing at 60 °C for 45 s and elongation at 72 °C for 30 s, concluding with a final elongation step at 72 °C for 5 min. The PCR products were checked by agarose gel electrophoresis. Pyrosequencing was performed on a PyroMark Q96 MD system (Qiagen) with PyroMark Gold Q96 reagents (Qiagen) using the primers MGMT-pyro1: TGGTGAGTGTTTGGGT and MGMT-pyro1R: CCAAACACTCACCAAAT. For the sample preparation process, 5–10 μl of PCR product were mixed with streptavidin-coated sepharose beads, followed by strand separation and washing with the vacuum prep tool (Qiagen). The bead-bound ssDNA was then mixed with 5 μl of sequencing primer (0.5 μM) per well and annealed by heating to 80 °C for 2 min and subsequent cooling to room temperature. The sequencing results were analysed with PyroMark MD software (Qiagen).

### EGFR analysis

The *EGFR* status was determined using the Zytovision SPEC *EGFR*/CEN7 Dual Color Probe FISH assay (Zytovision, Bremerhaven, Germany). The green fluorochrome labeled *EGFR* probe hybridizes to the 7p11.2 region encompassing the *EGFR* region, the orange labeled CEP7 probe hybridizes to the alpha satellite centromere region of chromosome 7 (7p11.1-q11.1). Slides with probe mix were co-denatured at 60 °C for 1 h and next hybridized at 37 °C for 16–24 h on a ThermoBrite (Abbott Molecular, Abbott Park, IL, USA). Sample pretreatment and post-hybridization washes were performed according to the recommendation of the FISH probe manufacturer (Zytovision). Slides were evaluated using a fluorescence microscopy with orange, green and DAPI compatible filters. FISH signal counts for orange and green were counted for a total of at least 100 nuclei in the solid tumor area and an *EGFR*/CEP7 ratio was calculated. *EGFR* amplification was defined by an *EGFR*/CEP7 ratio ≥ 2.0. Polysomy with an *EGFR*/CEP7 ratio < 2.0 or an *EGFR*/CEP7 ratio ≥ 2.0 in less than 15% of the tumor cell nuclei was defined as no *EGFR* amplification [24].

### Statistical analysis

Overall survival (OS) was defined as the primary endpoint and was calculated from the day of first surgery to the date of death. OS was estimated by the Kaplan Meier method using the Logrank test. A *p*-value less than 0.05 was considered to be of statistical significance. Cox regression analysis was applied to determine the independent prognostic impact of the IDH mutation status, *MGMT* methylation status as well as pure histopathological diagnosis (AAI, GBM), age at diagnosis (dichotomized at 65 years), preoperative clinical status (KPS, dichotomized at 70%) and extend of resection (GTR, STR). The software OrignPro v9.5.1 (Northampton, MA) and JMP 14.2.0 (Cary, NC) were used for statistical analysis.

## Results

### Clinical characteristics

A total of 139 patients were identified fulfilling the inclusion criteria. The median age was 63 years (AA: 50 years, GBM: 65 years), the male to female ratio was 1.4 to 1 (AA: 1.3, GBM: 1.5). In summary, 103 patients received a gross total resection (GTR), 30 patients a subtotal resection (STR), 5 patients had only a biopsy taken and in one case no information about the extent of surgery was available. The median overall survival was 14 months (AA: 41 months, GBM: 13 months) and the median KPS prior to surgical resection was 70 (AA: 80, GBM: 70).

### Neuropathological and molecular characteristics

After histopathological assessment of all 139 cases, 116 tumors were graded as GBM (83.5%) and 23 as AA (16.5%). Sequencing revealed that 19 cases were IDH1 mutated, mostly R132H mutations (18 cases) and one R132C mutation. No *IDH2* mutations were detected while one case of GBM could not be sequenced for IDH mutations. Thirteen IDH mutations were found in AA (56.5%), 6 IDH mutations in GBM (5.2%). This resulted in a distribution of 13 AA IDH-mut, 10 AA IDH-wt, 6 GBM IDH-mut and 110 GBM IDH-wt.

In only 2 out of 139 cases the *MGMT* status could not be determined, both of them GBMs. In 63/137 cases (46%) the *MGMT* promoter was methylated while 74/137 cases (54%) an unmethylated *MGMT* promoter was found. A *MGMT* promoter methylation was found in 20 of the 23 analysable AA (87%), in 3/23 cases no *MGMT* methylation was seen (13%). In two GBM *MGMT* analysis was not possible, in 43/114 cases *MGMT* methylation was found (38%), while in 71/114 cases the *MGMT* promoter was not methylated (62%). In 18/19 of the AA and GBM with IDH mutation a methylated *MGMT* promoter was found (95%), in one case (an AA) no *MGMT* promoter methylation was detectable despite an IDH mutation (5%). *EGFR* analysis was successfully performed in 136/139 cases. In 78/136 cases (57%) no amplification, in 58/136 tumors (43%) an *EGFR* amplification was observed. In 53/114 GBM with a successfully determined *EGFR* status an amplification was observed (46%). An high level of *EGFR* amplification was identified in 5/10 (50%) of patients with an AA IDH-wt, thereby allowing the designation of a ‘*Diffuse astrocytic glioma, IDH-wildtype, with molecular features of glioblastoma, WHO grade IV’* according to cIMPACT-NOW update 3 [25]. None of the tumors with an *EGFR* amplification showed an IDH mutation. Within the group of 58 tumors with an *EGFR* amplification *MGMT* promoter methylation was evident in 24 cases (41%). In 37/76 cases without *EGFR* amplification *MGMT* promoter methylation was observed (49%) Table 1.

**Table 1:** Patients characteristics

	AA *n* = 23	GBM *n* = 116
Age in years		
Median	50	65
Range	25–74	26–81
Age (> 65, <= 65)	6, 17	63, 53
Gender (male/female)	13, 10 (1.30:1.00)	69, 47 (1.49:1.00)
Extent of resection		
GTR	14 (61%)	89 (77%)
STR	5 (22%)	25 (22%)
Biopsy only	4 (17%)	1 (1%)
N.d.	0	1 (1%)
KPS (> 70, <= 70, n.d.)	15 (65%), 6 (26%), 2 (9%)	42 (36%), 73 (63%), 0
IDH +, IDH -	13 (57%), 10 (43%)	6 (5%), 110 (95%)
MGMT+, MGMT-, MGMT n.d.	20 (87%), 3 (13%), 0	43 (37%), 71 (61%), 2 (2%)
IDH+ & MGMT+	12 (52%)	6 (26%)
IDH- & MGMT-	2 (7%)	71 (61%)
IDH+ & MGMT-	1 (4%)	0
IDH- & MGMT+	8 (35%)	37 (32%)
EGFR+, EGFR -, EGFR n.d.	5 (50%), 5 (50%), 0	53 (46%), 61 (54%), 3 (2%)

*AA* anaplastic astrocytoma, *GBM* glioblastoma, *GTR* gross total resection, *STR* subtotal resection, *n.d*. not determined, *KPS* Karnofsky Performance Scale, *IDH+ IDH1* or *IDH2* mutation, *IDH-* no *IDH1* or *IDH2* mutation, *MGMT+* promoter methylation, *MGMT-* no promoter methylation, EGFR+ amplification, *EGFR*- no amplification

### Survival analysis

**Fig. 1:**
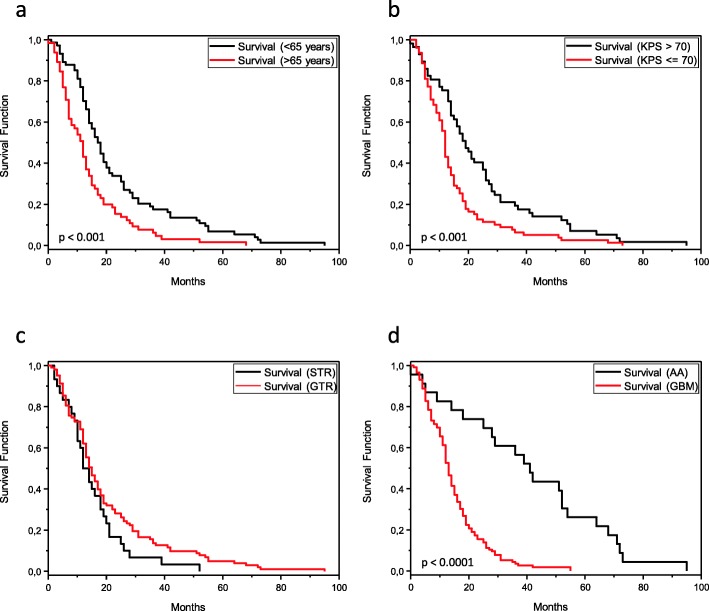
Kaplan Meier plots a series of 139 patients with malignant astrocytomas, which shows an association between (**a**) overall survival and an age below and above 65 years. **b** A significance is also found in the cohort between a Karnofsky Performance Scale (KPS) below and above 70. **c** The univariate analysis of the extent of tumor resection (EOR) did not show a significant difference, yet in multivariate analysis EOR appeared as an independent prognostic factor. **d** However, there is a clear association between the pure histopathological diagnosis of anaplastic astrocytoma (AA) and a glioblastoma (GBM)

In the univariate analysis of all patients with a malignant astrocytoma, age below 65 years (*p* <  0.001; Fig. 1a; Table 2) and a KPS above 70 (*p* <  0.001; Fig. 1b) were associated with a longer OS. However, no link between OS and sex (Fig. 1c) or GTR and STR was observed. Patients with the histopathological diagnosis of an AA performed significantly better regarding overall survival compared to patients diagnosed with a GBM (*p* <  0.0001; Fig. 1d).

**Table 2:** Survival characteristics of patients

		mOS in months		mOS in months	p
Age	< 65	17.5	> 65	12	< 0.001
KPS	> 70	19	< 70	12	< 0.001
Sex	M	15	F	13	n.s.
EOR	GTR	15	STR	13	n.s.
Histo	AA	41	GBM	13	< 0.0001
MGMT	MGMT+	20	MGMT-	12	< 0.001
MGMT AA	MGMT+	41,5	MGMT-	18	n.d.
MGMT GBM	MGMT+	15	MGMT-	12	n.s.
IDH	IDH+	51	IDH-	13	< 0.0001
IDH AA	IDH+	54	IDH-	16	< 0.0001
IDH GBM	IDH+	11	IDH-	13	n.s.
EGFR	EGFR+	14	EGFR-	14	n.s.
EGFR AA	EGFR+	9	EGFR-	42	< 0.05
EGFR GBM	EGFR+	14	EGFR-	12	n.s.
IDH/MGMT	IDH+/MGMT+	46,5	IDH+/MGMT-	64	n.d.
IDH/MGMT	IDH+/MGMT+	46,5	IDH−/MGMT+	15	< 0,00001
IDH/MGMT	IDH+/MGMT+	46,5	IDH−/MGMT-	12	n.s.
IDH/MGMT	IDH−/MGMT+	15	IDH+/MGMT-	64	n.d.
IDH/MGMT	IDH−/MGMT+	15	IDH−/MGMT-	12	n.s.
IDH/MGMT	IDH+/MGMT-	64	IDH−/MGMT-	12	n.d.

*mOS* median overall survival in months, *p* p-value by Logrank test with a value less than 0.05 indicating statistical significance, *Histo* histological diagnosis, *AA* anaplastic astrocytoma, *GBM* glioblastoma, *GTR* gross total resection, *STR* subtotal resection, *M* male, *F* female, *n.s.* not significant, *n.d.* not determined, *KPS* Karnofsky Performance Scale, *EOR* extent of resection, *IDH+ IDH1* or *IDH2* mutation, *IDH-* no *IDH1* or *IDH2* mutation, *MGMT+* promoter methylation, *MGMT-* no promoter methylation, *EGFR+* amplification, *EGFR*- no amplification

**Fig. 2:**
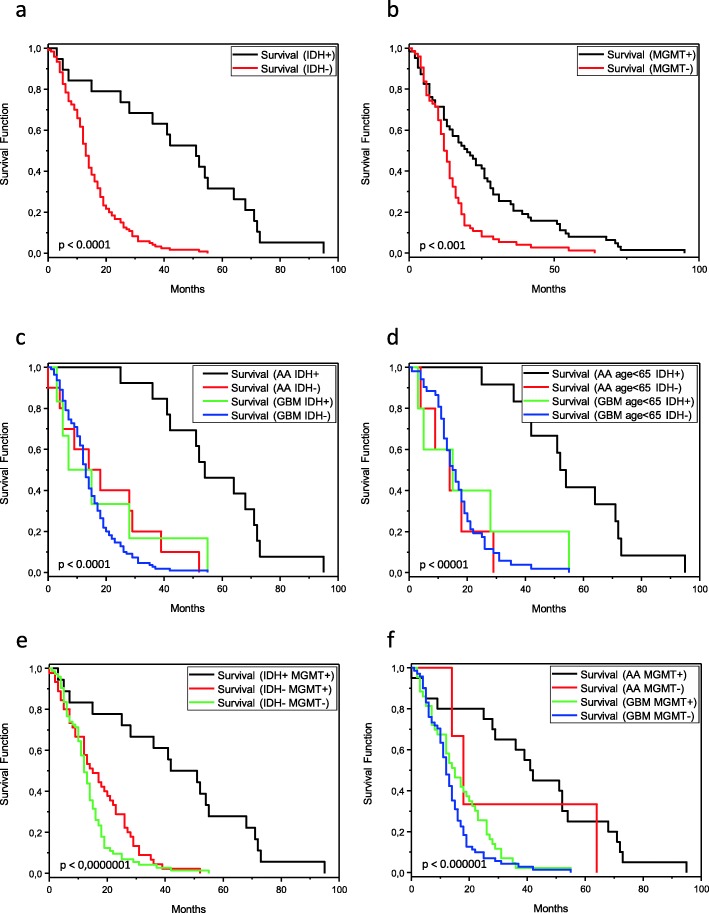
Kaplan Meier plots derived from 139 patients diagnosed with an anaplastic astrocytoma or glioblastoma. **a** There is an association between overall survival and IDH status and (**b**) between overall survival and *MGMT* status. **c** There is a significant effect in the combination of histopathological diagnosis and IDH status. Patients with anaplastic astrocytoma and IDH mutation show better overall survival than patients with anaplastic astrocytoma without IDH mutation and patients diagnosed with glioblastoma with/without IDH mutation. The overall survival of the patients of the latter three subgroups appears similar in the Kaplan Meier analysis. **d** The same significant effect regarding histopathological diagnosis and IDH status became evident when only patients were subject to analysis below the age of 65 years. **e** Combining IDH and *MGMT* status patients with a positivity for both markers performed better than those patients with a negative IDH status independent from the *MGMT* status. Because only one tumor showed the combination IDH+ and MGMT- this group was not plotted. **f** Analyzing the histopathological diagnosis in combination with the *MGMT* status patients with an anaplastic astrocytoma with *MGMT* promoter methylation showed a better overall survival than the other groups. However, only 3 patients with an anaplastic astrocytoma revealed a negative *MGMT* promoter status so no further conclusion could be drawn from this group. AA: anaplastic astrocytoma, GBM: glioblastoma, IDH+: *IDH1* or *IDH2* mutation, IDH-: no *IDH1* or *IDH2* mutation, MGMT+: promoter methylation, MGMT-: no promoter methylation

All patients with an AA or GBM demonstrated a significant longer OS in case of *MGMT* promoter methylation (*p* <  0.001; Fig. 2b; Table 2). Analysing OS in context of the *MGMT* status depending on histology, patients with *MGMT* promoter methylation and an AA showed the longest OS (median OS 41,5 months), while patients with an unmethylated *MGMT* status and a GBM had the shortest OS (median OS 12 months). Patients with *MGMT* promoter methylation and a glioblastoma showed an OS in between (median OS 15 months). Because only in three cases the combination of an unmethylated *MGMT* promoter with the diagnosis of an AA was found, no conclusions regarding this groups could be undertaken (median OS 18 months). Comparing only patients with a GBM in respect to *MGMT* status only a trend towards a better OS for those with promoter methylation was found (*p* = 0.08). Furthermore, no significant difference regarding the OS was seen between patients with an AA and a positive or negative *MGMT* status (*p* = 0.4). A clear difference in OS was identified between patients with an AA and a positive *MGMT* status and patients with a GBM and a positive *MGMT* status (*p* <  0.00001). In a pooled analysis of all four groups a clear prognostic significance of a methylated *MGMT* status was found (*p* <  0.000001; Fig. 2e).

All patients with a AA or GBM demonstrated significantly longer OS in case of an IDH mutation (*p* <  0.0001; Fig. 2a; Table 2). Furthermore, in the group of patients with AA IDH mutations were associated with a longer OS (*p* <  0.0001). However, the small number of 6 patients with an IDH-mutated GBMs allowed a statistical evaluation with only very limited validity. Next, we did not find a significant difference between patients with an AA IDH-wt and all GBM patients and subjects with a GBM IDH-wt. Patients with an AA IDH-mut had a prolonged OS compared to patients with a GBM IDH-mut (*p* <  0.05). In summary, the Kaplan Meier figure of all four groups more or less showed very similar plots for patients with GBM IDH-wt, AA IDH-wt and GBM IDH-mut compared to patients with AA IDH-mut. The differences in OS across the whole group of patients were highly significant (*p* <  0.0001; Fig. 2c). A similar effect was observed in a combined analysis of four subgroups as well when only patients younger than 65 years were analysed (*p* <  0.00001; Fig. 2f). Pooling all malignant astrocytic tumors with available IDH and *MGMT* status three molecular groups allowed further analysis regarding OS: IDH-mutated and *MGMT* methylated, IDH-wildtype and *MGMT* methylated and IDH-wildtype and *MGMT* not methylated. Because only one tumor showed the molecular signature IDH-mutated and *MGMT* not methylated this variant was not further evaluated. A clear prognostic difference between the molecular subgroups IDH-mutated and *MGMT* methylated versus IDH-wildtype and *MGMT* methylated was observed (*p* < 0,00001). No significance was detected between the groups IDH-wildtype and *MGMT* methylated versus IDH-wildtype and *MGMT* not methylated but a trend for a longer OS in the first molecular group was seen (*p* = 0.06). Combining the analysis of all 3 molecular subgroups a clear association was found as well (*p* <  0,0000001, Fig. 2d). No link between *EGFR* amplification and OS was found by analysing all tumors, all GBM and all AA IDH-wt, respectively (Table 2). However, comparing AA with and without EGFR amplification a prognostic effect was evident (*p* <  0.05).

The multivariate analysis showed an independent prognostic significance of the variables gross total resection (GTR) (p <  0.05), anaplastic astrocytoma (*p* <  0.0001) and IDH mutation (*p* < 0.0001) when all patients were evaluated. The presence of a methylated *MGMT* promoter remained insignificant as well in the multivariate analysis. Age at diagnosis below 65 and a KPS status of 70 or more were identified as prognostic factors in the univariate analysis. However, the multivariate analysis did not indicate that they are independent prognostic factors. If patients who only received a biopsy (*n* = 5) were excluded, the variables gross total resection (*p* < 0.05), anaplastic astrocytoma (*p* < 0.0001) and IDH mutation (p < 0.05) remained as independent prognostic factors.

## Discussion

The observation that the IDH status in patients with malignant astrocytic tumors has a significant prognostic relevance [8–10, 26–28] has led in recent years to significant modifications in the classification [6] and treatment of these tumors [21]. For instance, the authors of the current EANO guideline [21] recommend radiotherapy for patients with AA and an IDH mutation, followed by temozolomide or PCV-based chemotherapy. Patients with AA IDH-wt, on the other hand, are recommended to receive radiotherapy alone if their *MGMT* status is negative, combined radiochemotherapy followed by temozolomide treatment if the *MGMT* methylation status is positive. The IDH status, on the other hand, has no consequences with regard to therapy recommendations for patients with GBM who are to be treated at an age below 70 years according to the Stupp protocol [22]. Interestingly, however, there are clear studies that provide a different therapy recommendation for patients with AA with and without IDH mutation [8], but not for the recommendation to treat patients with AA IDH-wt (and *MGMT* promoter methylation) identically to GBM patients. However, the underlying observation that these are patients of the same genetic entity with a similar prognosis was based on a pooled data set of NOA-04 and GGN [10]. At that time, the patients under investigation were treated by a regimen based solely on the histological diagnosis, which differed between WHO grade II and WHO grade IV gliomas. The aim of this study is now to assess whether the overall survival of patients with AA IDH-wt is comparable to that of GBM patients with identical therapy according to the Stupp protocol.

The clinical and molecular characteristics of the patients analysed in this cohort are within the range of established data on these tumor entities. The CBTRUS report for the years 2011–2015 states a median age of 65 years for GBM patients and 50 years for patients with AA [29]. The median average age of the patients examined here was 65 years and 53 years, respectively. The ratio of male to female was 1.36:1.00 for GBM (presented cohort 1.49:1.00) and 1.20:1.00 (1.30:1.00) [29]. In the present study, 57% of patients with AA and 5% of patients with GBM had IDH mutations, while 60 and 7.2%, respectively, were found in a comparable study [10]. In 87% of patients with AA (reference: 55%) and in 37% (reference: 47%) of GBM patients *MGMT* promoter methylation was found [10]. While we found an *EGFR* amplification in 46% of the patients with a GBM, a frequency of 35–45% is reported in the literature [6]. We identified *EGFR* amplification in 50% of patients with AA. Other studies reported a lower rate of less than 10% [30]. However, it must be taken into account that with the low number of cases of 23 patients with AA investigated here, a sampling error seems conceivable as explanation. Established risk factors regarding overall survival for patients with malignant diffusely infiltrating astrocytomas are the histopathological criteria of necrosis and vascular proliferation, the age of the patient, the extent of resection, the KPS as well as molecular characteristics like *MGMT* and IDH status [21]. In the univariate analysis, we were able to confirm all these characteristics as risk factors in the cohort examined here except for the extent of resection (see Figs. 1 and 2). The extent of resection was subjectively assessed by the surgeon immediately following the operation. However, neuroradiological postoperative quantification was not performed. Accordingly, it seems conceivable that the subjective assessment by various neurosurgeons did not lead to consistent data, so that the assessment of the extent of resection was erratic and could therefore not be identified as a risk factor. In summary, however, it can be concluded that the series of patients examined is representative for the question addressed.

Several interesting observations result from the molecular data of the cohort of patients with malignant astrocytic tumors investigated here. As one of the most important observations it could be documented that patients with AA IDH-wt showed a similar overall survival as patients with GBM IDH-wt despite combined radiochemotherapy. With regard to this aspect it could be expected that a more aggressive therapy according to the Stupp protocol would result in a prolonged overall survival for patients with AA IDH-wt. In the comparative study [10], only 20% of these patients received combined radiochemotherapy, while in the cohort analyzed here all patients diagnosed with AA IDH-wt were treated accordingly. Despite this more aggressive therapy, no increase in overall survival was documented. On the other hand, the survival plot line of patients with AA IDH-wt was even closer to that of patients with GBM in the Kaplan Meier graph (Fig. 2c). Various conclusions can be drawn from this observation. Firstly, it can be concluded that AA IDH-wt actually genetically correspond to GBM IDH-wt, since patients with these tumors show a very similar overall survival under comparable therapeutic conditions. Secondly, the question also arises whether the additional cytotoxic side effects of a more aggressive therapy justify the lack of increase in overall survival for patients with AA IDH-wt. However, it must be critically noted that this conclusion is based on a rather small number of patients in the present study. Prospective studies with a larger number of patients are necessary to generate sufficient data regarding this issue.

Furthermore, it is remarkable that in terms of overall survival, patients with GBM IDH-mut do not differ from patients diagnosed with GBM IDH-wt or AA IDH-wt. In the corresponding comparative study [10], the Kaplan Meier plot showed a plot line of these patients above the line of patients with AA IDH-wt. However, no significance was apparent in the comparative study either. On this basis, it was discussed to potentially treat patients with a GBM IDH-mut less aggressively than patients with a GBM IDH-wt. The data presented here argue rather against such a suggestion to modify the established therapy according to the EANO guideline [21]. However, it must be emphasized that a small number of patients with this genetic alteration is the basis of the conclusion.

With regard to the question whether the established histopathological criteria for grading of malignant astrocytic tumors in the IDH age remain important under identical therapy, it can be concluded from the available data that this is still the case to some extent. There is a significant difference in OS between patients with AA IDH-mut and GBM IDH-mut. Therefore, it can be concluded for this group of malignant astrocytomas that the detection of necrosis and vascular proliferation remains important for the evaluation of the prognosis. A different picture emerges when we consider patients with AA and GBM that show no IDH mutation. The corresponding Kaplan Meier plots for OS of these patients are very similar and no significant difference can be shown. Accordingly, it can be concluded from the data that for patients with malignant diffusely infiltrating astrocytomas without IDH mutation, the histopathological grading criteria necrosis and vascular proliferation are only of minor importance. It should be noted that recently homozygous *CDKN2A* deletions were identified as prognostic markers for malignant astrocytic tumors with IDH mutation [31]. This molecular marker was not evaluated in the present study. However, an analysis of the *CDKN2A* status would be of interest for a subsequent study in order to evaluate its significance in the context of classical histopathological grading criteria.

The frequently published prognostic data on patients with AA IDH-wt indicate that these tumors are genetically GBM IDH-wt [8, 10, 26–28]. While this hypothesis is probably true for the largest number of such tumors, it cannot be excluded that a fraction of histopathologically defined AA corresponds indeed to another entity. For example, an unrepresentative biopsy of an anaplastic ganglioglioma, an anaplastic pleomorphic xanthoastrocytoma or a GBM with an H3F3A G34R/V mutation may also appear as a diffuse infiltrating AA IDH-wt. If in this combination only the absence of an IDH mutation in tumors with a malignant astrocytic morphology that do not exhibit necrosis and/or vascular proliferation were determined as definition criteria, the above-mentioned prognostically more favorable tumors would incorrectly be assigned to a WHO grade IV. For this reason, cIMPACT-NOW Update 3 requires the detection of an *EGFR* amplification, a *TERT* promoter mutation or a complete gain of chromosome 7 combined with a complete deletion of chromosome 10, in addition to the two criteria ‘*diffuse astrocytoma’* and ‘*no IDH mutation’*, in order to be able to establish the diagnosis ‘*Diffuse astrocytic glioma, IDH-wildtype, with molecular features of glioblastoma, WHO grade IV’* [25]. For this reason, we determined the *EGFR* status of all patients. Indeed, an *EGFR* amplification was found in 5/10 anaplastic AA IDH-wt, so that these tumors can subsequently be assigned to a WHO grade IV according to cIMPACT-NOW update 3. Due to the remaining very small number of 5 AA IDH-wt without *EGFR* amplification, we refrained from further statistical evaluation.

While methylation of the *MGMT* promoter had a clear prognostic significance in the entire cohort of malignant astrocytic tumors, isolated analysis of patients diagnosed with GBM revealed only a trend in this direction. Most studies document a prognostic significance of *MGMT* promoter methylation for GBM in the context of combined radiochemotherapy [9, 17]. Other publications, however, did not report a prognostic significance of *MGMT* promoter methylation [32–34]. Various reasons can be debated for the discrepant observation. Tthere are no generally accepted and standardized assays for *MGMT* analysis, so that especially in the context of different analyzed CpG sites, distinct results can emerge. The *MGMT* assay we use covers 14 CpG sites [23], which also cover the region, which are mostly given as reference [17]. Accordingly, we assume that the assay we use sufficiently determines the *MGMT* status. Furthermore, it is discussed that a different material quality (frozen tissue versus FFPE tissue) can lead to different *MGMT* results. In summary, however, the most likely explanation for our observations is a slight sampling error which leads to the lack of significance while still a trend can be observed.

The present study has various weaknesses. The number of 139 patients diagnosed with malignant astrocytoma included in the study appears to be rather limited, considering that only 13 patients diagnosed with an AA IDH-mut, 10 patients diagnosed with an AA IDH-wt and 6 patients diagnosed with a GBM IDH-mut are covered. Furthermore, it is a retrospective analysis. Accordingly, a prospective study would be recommended, which should include a significantly larger number of patients with the above-mentioned diagnoses. The CATNON study is expected provide a large dataset when IDH data become available [35]. However, no GBM patients are covered in the CATNON study, so that no conclusions can be drawn from these study data regarding the histopathological grading criteria for malignant astrocytic tumors. Another deficit of the study presented here are incomplete clinical data regarding progression-free survival (PFS), which did not allow sufficient analyses. Especially in the context of identical therapeutic conditions, interesting data could have been generated by the correlation of PFS and histopathological and molecular markers. In this respect, the CATNON study may also provide further information when IDH data will be available [35]. Furthermore, we always used preoperative imaging information to determine contrast-enhancing regions to ensure that the most malignant tumor regions were resected in case of subtotal resection or biopsy. However, we do not have adequate postoperative imaging in all cases so we cannot exclude the possibility that a few AA might be undergraded GBM. In addition, even in case of totally resected tumors we cannot rule out the possibility that we missed necrotic areas or microvascular proliferates in some tumors diagnosed as AA, due to non-representative sampling of the tissue for histological processing.

In summary, the study presented here shows that patients diagnosed with an AA IDH-wt have basically the same prognosis as patients diagnosed with a GBM IDH-wt when treated according to the Stupp protocol. Therefore, the concept of the current WHO classification to stratify patients with malignant astrocytomas according to the IDH status can be confirmed [6]. The data generated here can also be quoted to confirm the concept of cIMPACT-NOW Update 3 that patients diagnosed with IDH wild-type AA correlate prognostically with WHO grade IV tumors [25]. In the absence of an IDH mutation, the established histopathological gradation parameters necrosis and vascular proliferation indeed lose their prognostic significance even under similar therapeutic conditions of a combined radiochemotherapy. However, if IDH mutations can be detected in such tumors, the presence of necrosis and/or vascular proliferation still allows further prognostic differentiation according to the results of the study presented here.
